# Molecular identification of a root apical cell-specific and stress-responsive enhancer from an *Arabidopsis* enhancer trap line

**DOI:** 10.1186/s13007-019-0393-0

**Published:** 2019-01-31

**Authors:** Lei Zhang, Li-Na Qin, Zi-Rui Zeng, Chang-Zheng Wu, Yuan-Yong Gong, Lai-Hua Liu, Feng-Qiu Cao

**Affiliations:** 1 Key Laboratory of Plant-Soil Interaction, MOE, Center for Resources, Environment and Food Security, College Resources and Environmental Sciences, China Agricultural University, Beijing, 100193 China; 2 Zhaoyuan Agricultural Technology Extension Centre, Zhaoyuan, 265400 Shandong China; 3 Key Laboratory of Cotton and Rapeseed, Ministry of Agriculture, The Institute of Industrial Crops, Jiangsu Academy of Agricultural Sciences, Nanjing, 210014 China; 4 Shanghai Centre for Plant Stress Biology of Shanghai Institutes for Biological Sciences, Chinese Academy of Sciences, Shanghai, 201602 China

**Keywords:** Enhancer trap line, Enhancer, Root apex specific expression, GFP and GUS, Abiotic stimuli

## Abstract

**Background:**

Plant root apex is the major part to direct the root growth and development by responding to various signals/cues from internal and soil environments. To study and understand root system biology particularly at a molecular and cellular level, an *Arabidopsis* T-DNA insertional enhancer trap line J3411 expressing reporters (GFP) only in the root tip was adopted in this study to isolate a DNA fragment.

**Results:**

Using nested PCR, DNA sequencing and sequence homology search, the T-DNA insertion site(s) and its flanking genes were characterised in J3411 line. Subsequently, a 2000 bp plant DNA-fragment (E_rtip1_) upstream of the insert position of the coding T-DNA was in silico analysed, revealing certain putative promoter/enhancer *cis*-regulatory elements. Cloning and transformation of this DNA fragment and its truncated segments tagged with or without 35S minimal promoter (35Smini), all of which were fused with a *GFP* or *GUS* reporter, allowed to detect GFP and GUS expression mediated only by E_rtip1_ + 35mini (P_Ertip1+35Smini_) specifically in the *Arabidopsis* root tip region. The P_Ertip1+35Smini_ activity was further tested to be strong and stable under many different growth conditions but suppressed by cold, salt, alkaline pH and higher ammonium and phosphorus.

**Conclusion:**

This work describes a promising strategy to isolate a tissue-/cell-specific enhancer sequence from the enhancer trap lines, which are publically available. The reported synthetic promoter i.e. P_Ertip1+35Smini_ may provide a valuable and potent molecular-tool for comprehensive investigation of a gene function related to root growth and development as well as molecular engineering of root-architectural formation aiming to improve plant growth.

**Electronic supplementary material:**

The online version of this article (10.1186/s13007-019-0393-0) contains supplementary material, which is available to authorized users.

## Background

One of the most important biological functions of a root system is to effectively explore soils for water and nutrients ensuring plant adaptive growth. There are three major processes involved in the development of root system: the cell division at the primary root (PR) meristem to add new cells enabling root growth; the generation of lateral roots (LR) to increase the exploratory capacity of the root system; the enlargement of the total surface of PR and LRs through root-hair growth [1]. Such processes are known to be greatly affected by varied environmental factors including nutrients, water, pH and temperature, etc., thus conferring a high degree of morphological-plasticity of root growth in adaptive response to often changed environmental cues [2]. Regarding the morphological plasticity, for instance, a spectrum of responses at physiological and cellular levels occurred when the roots are under water stress [3]; Arabidopsis LR development was strongly promoted to the soil with rich nitrate (NO_3_^−^) [4]; a profound effect of the combined supply of phosphorus and magnesium on the development of root system morphology was observed in Arabidopsis via auxin signaling, which regulates the elongation and directional growth of the primary root [5].

Morphologically and functionally, along the (primary) root axis, four zones can be divided: root cap, meristematic region, elongation-and maturation-zone. The root apex or tip represents the most critical part required for sensing and adaptively responding to environmental stimuli [6]. A study with Arabidopsis showed that the treatment with 5 mM KNO_3_ could inhibit the primary root growth by 30–100% in 3 days, depending on an effect associated with a significant increase in auxin concentration at the root apex [7]. Recently, Medici et al. [8] described that a reasonable molecular gate integrating phosphate (Pi)- and NO_3_^−^-signalling via AtNIGT1/HRS1 actions might exist at the Arabidopsis root apex, regulating the response of root system growth to environmental Pi and NO_3_^−^. Besides, there is also an evidence that exogenous l-glutamate at micromole concentrations can act as a highly specific signal molecule sensed by the root tip to modify root growth and branching [9]. However, physiological and molecular determinants necessary for the architectural formation of root system, directed by the root apex in response to varied environment cues, are largely unknown.

To evaluate and/or manipulate a function of an interested genetic component (or gene) involved in the root growth at cellular and molecular levels, the application of a particular promoter driving the gene expression in root cell-type-specific organs (e.g. the root tip) should be a promising strategy. Methodologically, it has been documented that a reporter- or marker protein-based enhancer-trap line (e.g. consisting of GAL4/GFP system) would provide one of the most powerful means for the exploration of a biological event(s) associated with a gene of interest in a cell-specific manner [10]. Basically, in the GAL4/GFP enhancer trap system, a given T-DNA is introduced randomly into a host genome; as the T-DNA integrates downstreamly near an enhancer-dependent or -activated promoter, the activity of the promoter and/or enhancer could be detected by the visualization of the green fluorescent signals derived from GFP, whose expression is GAL4-responsive [11]. To date, *GAL4/GFP* enhancer-trap lines of some plant species (e.g. Arabidopsis and rice) were created and publically available [11, 12]. The use of these transgenic lines greatly favoured many excellent studies elucidating biological processes at organ/tissue/cell levels [12–14]. An additional significant contribution of such enhancer-trap lines to biological study is that some promoters with different cell-specific activities were molecularly identified based on finding of cell-type specific genes [15–17], allowing a precise assessment and manipulation of a gene function with a cell- and developmental-specificity.

The precise temporal-spatial regulation of gene expression is pivotal for the prosperous production of highly-specialized organs/cells and their abilities to respond to environmental signals [18]. At a molecular level, this is greatly completed by the activation and/or repression of the related *cis*-regulatory elements (e.g. transcriptional enhancers and silencers) at the correct place and time [19, 20]. Generally, the promoter together with its up- or down-stream distal sequence (e.g. enhancer) is crucial *cis*-components required to control the expression of its target gene(s) [21], contributing to the regulation of plant growth and development. Thus, the isolation and subsequent application of the valuable promoter and/or enhancer allow a molecular manipulation of plants by miss- or over-expression of a functional gene of interest [22]. As documented, despite the wide application of strong and constitutive promoters (e.g. ubiquitin gene promoter and CaMV 35S RNA promoter), they triggered the gene overexpression in all tissues might impair the host plant growth and development [23, 24]. In contrast, a tissue-specific promoter can accurately control the transcription within a given plant part, possibly avoiding undesirable or negative effects from expressing a foreign gene [25]. Previously, although an approach through finding tissue-specific expressing genes was widely used to isolate related promoters from Arabidopsis, rice, sweet potato and soybean [26–29], this method seems fairly tedious and inefficient because of the requirement of experimental identification and confirmation of cell-type specific expression patterns of the genes [29], and some tissue-specific promoters isolated based on this way might have a low activity or specificity [29]. Regarding a promoter with its activity confined only to the root apex/tip, related publications are hitherto very limited, most probably due to people’s interest in its patent protection.

We report here a simple, direct and precise method for the isolation of a putative enhancer (E_rtip1_) for the root apex-specific transcription from a *GAL4/GFP* enhancer trap line J3411. The activity of the enhancer (fused with or without a 35S minimal promoter) was monitored by the expression and detection of reporter proteins (i.e. GFP and GUS) in Arabidopsis transgenic plants, showing its strong and specific action only in the root apex/tip zone. Furthermore, to evaluate the stability of this enhancer activity, GFP-indicated fluorescent signals were tested under varied growth conditions, revealing that the enhancer-facilitated reporter expression was strongly and rapidly suppressed by certain external stimuli. Thus, such a cell/tissue-specific enhancer and its synthetic promoter (like P_Ertip1+35Smini_ constructed in the work) should provide a valuable and potent molecular tool to favour the intensive investigation of root system biology as well as manipulation of root growth and function.

## Methods

### Plant materials and growth conditions

A *GAL4/GFP* line J3411 (Arabidopsis C24 background) was obtained from the Haseloff and Poethig collections, (http://data.plantsci.cam.ac.uk/Haseloff/tools/gal4system/page138.html). Transgenic plants harbouring putative promoters/enhancers fused with *GFP* or *GUS* were generated in this work (see “Generation of transgenic lines”).

For Arabidopsis aseptic growth, surface-sterilized seeds were germinated and cultivated vertically for 7 d on the basic medium i.e. a half-strength MS agar (0.8%)-medium (containing 1% sucrose and 0.5 mM NH_4_NO_3_) in a growth room (19–22 °C, 16 h/8 h light/dark period, 120 μmol m^−2^ S^−1^ light intensity); thereafter, seedlings were transferred to the basic medium (except for N- or P-treatment) plate for further 1 d growth under 20 different treatments as shown below: IAA (Indo-3-acetic acid, 60 nM), ABA (Abscisic acid, 200 nM), GA (Gibberellic acid or Gibberellin, 500 nM), ACC (1-Aminocyclopropane-1-carboxylic acid, 500 nM), 6-BA (6-Benzylaminopurine, 100 nM), l-Glu (0.5 mM), l-Leu (0.5 mM), l-Lys (0.5 mM), l-Met (0.5 mM), pH (4.5 and 8), P (phosphorus, high-2.5 mM, low-50 μM; in the form of KH_2_PO_4_), NH_4_^+^ (high-10 mM, low-10 µM; in the form of (NH_4_)_2_SO_4_), NO_3_^−^ (high-10 mM, low-10 µM; in the form of KNO_3_), AlCl_3_ (50 μM), Salt (NaCl, 80 mM), and cold (4 °C). Above chemical solutions were filter-sterilized and added to the autoclaved agar-medium (at about 60 °C). 7-d-old plants were transferred to the basic medium and grown for 1 d were used as a reference (CK) in the GFP assay. Except for those treated with cold (4 °C), all plants were grown under normal growth conditions as described above. Exception of pH treatment (4.5 and 8 adjusted respectively by using HCl or KOH), the medium pH was set to 5.8 by KOH. Under conditions of cold, salt, higher-pH, -salt, -NH_4_^+^ and -P, GFP expression in the transgenic plants harbouring P_Ertip1+35Smini_:*GFP* was particularly measured at different time point within 24 h (i.e. 0 h, 1 h, 6 h, 24 h).

To check the GFP/GUS expression in other upper-part tissues/organs (e.g. flower and silique), homozygous lines harbouring all individual truncated promoter/enhancer versions fused with GFP/GUS were cultivated in pot-soils for 70 d in the growth room.

### Detection of the T-DNA insert position and its flanking sequence/gene

Genomic DNA was isolated from 2-week-old J3411 seedlings (around 100 mg) by using CTAB (cetyltrimethyl ammonium bromide) extraction buffer (1% CTAB, 100 mM Tris–HCl (pH 8.0), 20 mM EDTA (pH 8.0), 1.5 M NaCl and water) and was then precipitated with isopropanol and washed with 70% alcohol. The Nest PCR (according to the protocol from http://signal.salk.edu/T-DNArecovery.pdf) was conducted with degenerate primers and a set of nested primers designed from the T-DNA left border (Additional file 1: Table S1). The Nest-PCR products were subsequently cloned into pGEM-T vector (Promega) and sequenced. Sequence homology search was carried out by using BLAST in the NCBI or TAIR (www.ncbi.nlm.nih.gov; https://www.arabidopsis.org/Blast/index.jsp).

### Sequence motif prediction

The sequence motifs or *cis*-regulatory elements in the putative promoter/enhancer E_rtip1_ were inspected using PLACE and PLANTCARE [30, 31]. The predicted important *cis*-regulatory elements are listed in Additional file 2: Table S2.

### Generation of transgenic lines

The putative promoter/enhancer E_rtip1_, E_rtip1_ + 35Smini, E_rtip2_, E_rtip2_ + 35Smini and E_rtip3_ (Fig. 2a) were PCR-amplified using specific primers (see Additional file 1: Table S1). All primers contain the *Spe*I site. PCR products were digested by *Spe*I and cloned into a plant expression vector pBI101-GUS and pBI101-GFP using compatible *Xba*I/*Spe*I cohesive ends, yielding constructs termed here: *E*_*rtip1*_:*GFP* or :*GUS*, *E*_*rtip1*_ + *35Smini*:*GFP* or :*GUS*, *E*_*rtip2*_:*GFP* or :*GUS*, *E*_*rtip2*_ + *35Smini*:*GFP* or :*GUS* and *E*_*rtip3*_:*GFP* or :*GUS*. Arabidopsis (Col-0) was transformed by floral dipping into a cell suspension (OD_600_ = 0.61) of agrobacterium strain GV3101 consisting of the above constructs. Plant transformants were selected by kanamycin resistance (50 μg L^−1^); at least two independent homozygous lines harbouring the above individual constructs were created in the T_2_ or T_3_ generation for experimental use. Exact numbers of independent transgenic lines generated for each of the five constructs are described in Additional file 3: Table S3. Three or six lines harbouring respectively *E*_*rtip1*_ + *35Smini*:*GUS* or :*GFP* show in fact their corresponding reporter expression only in the root tip (Additional file 3: Table S3 and Additional file 4: Fig. S1).

### GUS histochemical staining and GFP microscopic observation

For the detection of β-glucuronidase (GUS) activity, plant tissues were vacuum-infiltrated (for 10 min) and incubated further for 35 h at 37 °C in a staining solution (25 mM sodium phosphate buffer at pH 7.0, 10 mM EDTA, 0.5 mM ferricyanide, 0.5 mM ferrocyanide, 0.1% Triton X-100, and 2 mM X-Gal i.e. 5-bromo-4-choro-3-indolyl-β-d-glucuronide cyclohexylamine salt). Thereafter, tissues were washed with 75% ethanol twice over a 24 h period to remove chlorophyll from leaves or flowers. Samples were visualized under a microscope (BX51, Olympus, Japan).

For the observation of GFP localization, whole seedlings or tissues were mounted in water or 30% sucrose under a glass coverslip, and GFP signals were scanned with an energy excitation at between 488 and 535 nm by confocal laser scanning microscope (OLYMPUS FluoView™ FV1000, Japan). Brightness and contrast pictures were adjusted using the Olympus FV1000 Viewer software.

The intensity of green fluorescence photographed by a fluorescence microscope-camera device (BX51, Olympus, Japan) was quantified using ImageJ (https://www.youtube.com/watch?v=nLfVSWcxMKw%26lc=UgjP3p6wpnEcOXgCoAEC). Every picture was taken at the same exposure time (i.e. 20 ms), where pixel values range from 0 to 255. The value of a non-fluorescing root image was taken as a background intensity.

### Statistical analysis

The data are given in the form of a mean value with a standard deviation of replicates. Statistical test was performed using the statistical software program SPSS version 16.0 (Beijing, China). Significant differences between treatments were determined by one-way analysis of variance (ANOVA), and post hoc comparisons were done using Tukey’s multiple range test at P < 0.05.

## Results and discussion

### Identification of a genomic region involved in GFP specific-expression in the root apex of an enhancer trap line

**Fig. 1:**
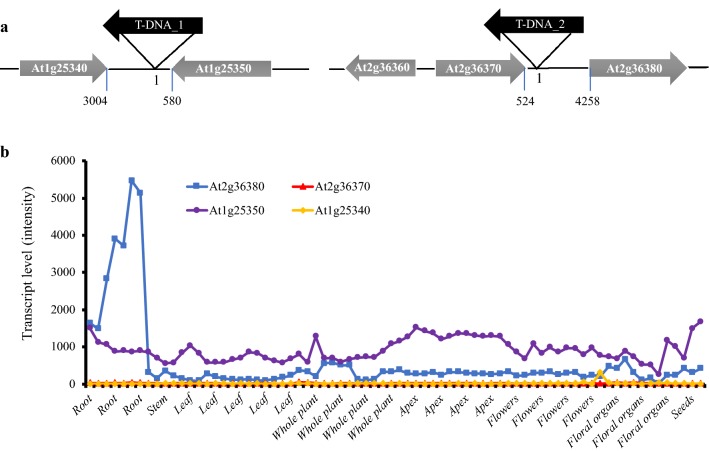
Identification of T-DNA inserts in J3411 and inspection of expression patterns of the T-DNA-flanking genes. **a** Two T-DNA insertion sites were detected at two different positions in the genome of the enhancer-trap line J3411. In J3411 plants, GAL4/UAS-dependent green florescent protein-derived signal was shown in the root tip region (Fig. 3f). Nest PCR, DNA sequencing and sequence homologous BLAST search were performed to characterize the position(s) of the T-DNA insert and its flanking genes (see “Methods” section). Arrows indicate the coding direction. **b** Transcriptional patterns of the T-DNA flanking genes i.e. At1g25340, At1g25350, At2g36370 and At2g36380 in varied tissues or at different developmental stages. Only At2g36380 was shown to be highly transcribed in roots. Transcript data were extracted from AtGenExpress (http://jsp.weigelworld.org/expviz/expviz.jsp. [34])

To isolate a putative root tip-specific promoter or enhancer, we inspected T-DNA-containing G*AL4*-*VP16*/*UAS*-*GFP* enhancer trap plants in the “Haseloff” database and obtained a valuable line (i.e. J3411) (http://data.plantsci.cam.ac.uk/Haseloff/tools/gal4system/page138.html), where GAL4-dependent GFP occurrence is specifically restricted to the root tip area (also see Fig. 3f). Since the T-DNA of this enhancer trap system harbours only a CaMV 35S RNA minimal promoter (containing TATA-boxes), which is not strong enough to drive the transcription of *GAL4* and further *GFP*, unless the T-DNA is integrated proximally to an enhancer or a promoter of a certain plant gene [14]. Thus, we applied nested PCR, DNA-sequencing as well as sequence homology search to characterize the position(s) of the T-DNA insert(s) in the J3411 genome and the genes flanking each insert(s). Resulting data indicated that there were two T-DNAs introduced respectively in the chromosome 1 and 2 of J3411 plant (Fig. 1a). The first T-DNA insert (termed here as T-DNA_1) is located between the coding sequences of At1g25340 (position 8885020-8886777, for a transcription factor MYB116 with deduced 283 amino acid residues) and At1g25350 (position 8889018-8894306, for a putative glutamine-tRNA ligase with 800 amino acids but without detailed functional description); and the second T-DNA (i.e. T-DNA_2) is flanked by the coding region of At2g36370 (position 15247768-15252980, for a predicted ubiquitin-protein ligase consisting of 940 amino acids) and At2g36380 (position 15257418-15263808, for a ABCG-transporter with putative 1453 amino acids, contributing to a defense against necrotrophic pathogens by mediating the secretion of camalexin [32]. This result is partially consistent with that reported by Radoeva et al., in which only one T-DNA (corresponding to the above stated ‘T-DNA_2’) was detected in J3411 by using the TAIL-PCR method [33]. Moreover, sequence alignment implied that the first T-DNA was coding in the opposite or same direction respectively to the flanking gene At1g25340 or At1g25350 (Fig. 1a), and the second was coding in the opposite direction to both the flanking genes i.e. At2g36370 and At2g36380 (Fig. 1a). Surveying gene expression patterns in public microarray databases [34] revealed that among the above four genes, only At2g36380 was highly transcribed in root tissues during plant development (Fig. 1b). Such data therefore proposes that the 4258 bp intergenic sequence (i.e. from T-DNA_2 insert site to the coding start of At2g36380, Fig. 1a) might contain a promoter and/or enhancer region to regulate the expression of both *GAL4/GFP* and At2g36380 in the root tip/apex.

### Certain important *cis*-acting regulatory elements are predicted in the 2-kb plant intergenic region upstream of the coding T-DNA_2

**Fig. 2:**
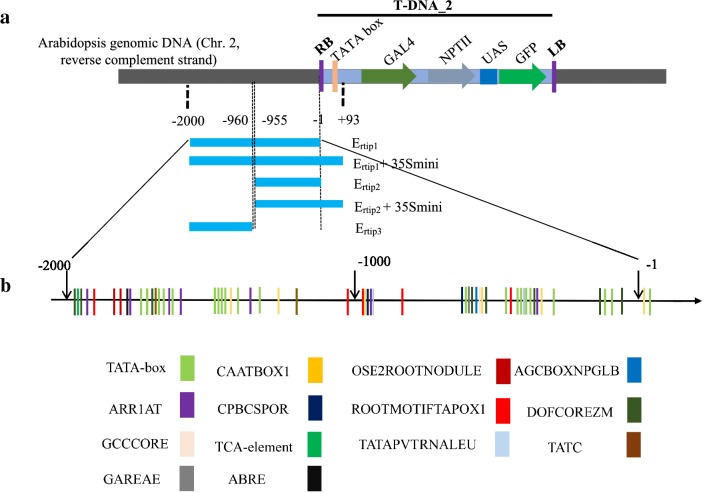
Schematic diagrams of five truncated versions and predicted important *cis*-elements of putative root-tip specific promoters/enhancers. **a** Designing of to-be-cloned five different versions of putative root-tip specific promoters/enhancers from J3411 line. E_rtip1_, E_rtip1_ + 35Smini, E_rtip2_, E_rtip2_ + 35Smini and E_rtip3_, DNA fragments (with 2000 bp, 2093 bp, 955 bp, 1048 bp and 1040 bp) derived respectively from the plant genomic DNA 2000 bp upstream of the insert site of the coding T-DNA, the 2000 bp sequence plus 93 bp from the T-DNA across the right border and minimal 35S promotor TATA-box (termed here as 35S mini), 955 bp upstream of the insert site of the coding T-DNA, the 955 bp sequence tagged with 93 bp 35S mini, 2000 bp to 906 bp upstream of the insert site of the coding T-DNA. Arrows indicate the coding direction of genes (e.g. *GAL4* and *GFP*) in the T-DNA. **b** Prediction of *cis*-acting elements in a putative root-tip specific promoter/enhancer E_rtip1_. Sequence motif prediction was conducted using “PLACE” and “PlantCARE” online-service (see “Methods” section). OSE2ROOTNODULE, a sequence motif related to the promoter activated in infected cells of root nodules; AGCBOXNPGLB and GCCCORE, elements involved in the ethylene responsiveness; ARR1AT, a binding element of the response regulator ARR1; CPBCSPOR, dealing with cytokinin response; DOFCOREZM, for the binding of Dof proteins involved in tissue-specific transcriptional enhancement; TATAPVTRNALE, involved in transcription re-initiation; ROOTMOTIFTAPOX1, related to root-specific gene expression; ABRE, involving the ABA function; TCA, a cis-element dealing with salicylic-acid action; TATC and GAREAE, a binding domain related to the gibberellin responsiveness. Such critical *cis*-elements with their rough positions in the putative root-specific enhancer(s) are indicated using different color bars

To assess a possible root tip-specific activity of the T-DNA flanking region, based on our T-DNA mapping and inspection of flanking gene expression patterns (Fig. 1), a two kb plant DNA (named here E_rtip1_; Fig. 2b; containing more root-specific elements than the region between 2-kb and 4258-bp, see below description) upstream of the insertion site of the coding T-DNA_2 was PCR-amplified from J3411, sequenced and analysed for the presence of a putative promoter as well as *cis*-regulatory elements. Sequence similarity search via BLAST indicated that the isolated E_rtip1_ sequence from J3411 (ecotype C24) was identical to that from Col-0 in the Arabidopsis database (Chr.2, 15255323-15253324). In silico analysis using online-software from Plant CARE [30] and PLACE [31] allowed to predict several promoter-related *cis*-acting elements in the E_rtip1_ (Fig. 2, Additional file 2: Table S2), including the basal element TATA-box (occurring 20 times) [35], and *cis*-acting element CAAT-box (presence of 5 times) that was reported to be responsible for the tissue-specific promoter activity [36]. Interestingly, certain root tip-specific *cis*-acting regulatory domains were identified in the E_rtip1_, which includes two OSE2ROOTNODULE-domains and five ROOTMOTIFTAPOX1**-**boxes that is important for a strong expression in roots [37–39]; eight DOFCOTEZM elements required for the binding of Dof proteins involved in plant-/tissue-specific transcription enhancement [40] (Additional file 2: Table S2, Fig. 2). Moreover, some hormone-responsive *cis*-regulatory elements were also detected: AGCBOXNPGLB and GCCCORE (involved in the ethylene responsiveness), CPBCSPOR (related to the cytokinin action), ABRE (dealing with the ABA action), TATC and GAREAE (involved in the gibberellin responsiveness) (Additional file 2: Table S2). Using such data leads to a suggestion that the E_rtip1_ might be a promoter or enhancer or consist of at least certain important *cis*-elements contributing the root-tip specific expression of GFP in the J3411 line.

### E_rtip1_ exhibits no promoter activity but enhances a T-DNA-derived 35S minimal promoter action only in the Arabidopsis root apex

**Fig. 3:**
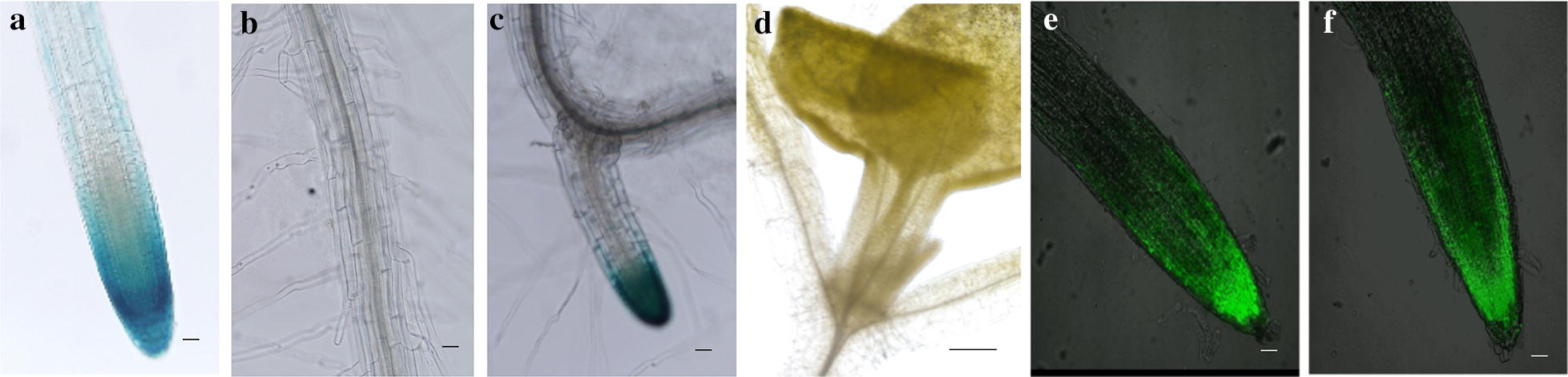
Detection of reporter expression in an Arabidopsis line only transformed with *E*_*rtip1*_ + *35Smini*:*GUS or* :*GFP*. Plant expression vectors harboring respectively E_rtip1_-, E_rtip1_ + 35Smini-, E_rtip2_-, E_rtip2_ + 35Smini- and E_rtip3_-fused *GUS* or *GFP* were constructed and their corresponding transgenic Arabidopsis (Col-0) lines were generated (till T2 or T3 generation, see “Methods” section). The enhancer/promoter activity was indicated by the GUS expression assayed via GUS-staining of transgenic plants grown on agar-plates (for 8 d) or pot-soil (over 2 months) (see “Methods” section). Except for the line transformed with E_rtip1_ + 35Smini:*GUS* (termed here as P_Ertip1+35Smini_:GUS), the GUS expression could not be detected in any tissue/organ of lines harboring respectively other individual constructs (data not shown). GUS-indicated enhancer/promoter activity was not detectable in flower organs and seeds of P_Ertip1+35Smini_:*GUS* plants (data not shown). **a** The primary root (PR). **b** A mature zone of the PR. **c** The lateral root. **d** Plant upper parts (e.g. leave and hypocotyl). Green fluoresce observation of the PR tip of P_Ertip1+35Smini_:*GFP* containing line (**e**) and J3411 (**f**). GUS, β-glucuronidase. A bar scale = 100 μm for **a**–**f**; the bar in 3 mm for **d**

To assess if and how E_rtip1_ could play a role in the event of a root tip-specific gene expression, the E_rtip1_ sequence and its two truncated segments (E_rtip2_, E_rtip3_) as well as E_rtip1_ and E_rtip2_ tagged with the 35S minimal promoter including TATA-box (termed here as 35Smini) from the T-DNA (i.e. E_rtip1_ + 35Smini (Additional file 5: Fig. S2), E_rtip2_ + 35Smini, Fig. 2) were cloned immediately before the *GUS*- and *GFP*-reporter into the promoter-lacking plant vectors (pBI101-GUS, pBI101-GFP, see “Methods” section), yielding constructs termed as E_rtip1_:*GUS/GFP*, E_rtip1_ + 35Smini:*GUS/GFP* (or P_Ertip1+35Smini_:*GUS/GFP*), E_rtip2_:*GUS/GFP*, E_rtip2_ + 35Smini:*GUS/GFP* and E_rtip3_:*GUS/GFP*. Transformation of these constructs into *Arabidopsis* (Col-0), and subsequent histochemical-assay and fluorescence microscopic-analysis allowed to effectively evaluate the transcriptional activity of E_rtip1_ and its related DNA segments in a tissue-/cell-specific manner. Of all transgenic lines, only plants carrying P_Ertip1+35Smini_:*GFP/GUS* showed a remarkable expression of the reporters. As presented in Fig. 3, the GUS activity was mainly detected only in the tip region of the primary- and lateral-root but not in other parts of the root, leaf and hypocotyl (Fig. 3a–d); the blue colour derived from GUS-staining did not occurred in flowers, siliques and seeds (data not shown). Further confocal microscopic observation revealed that strong green-fluorescence signals were confined also mostly to the root-apical meristermatic domain of the transgenic line introduced only with P_Ertip1+35Smini_:*GFP*, in agreement with that of visualized in J3411 (Fig. 3e, f. http://data.plantsci.cam.ac.uk/Haseloff/tools/gal4system/page138.html**)**, affirming the action of E_rtip1_ + 35Smini is specific to the root apex of *Arabidopsis*.

Since the activity of E_rtip1_ along or its truncated DNA fragments with or without the 35Smini in triggering *GUS/GFP* expression could not be detected in their corresponding transgenic lines (data not shown), the E_rtip1_ should be considered as a potent enhancer responsible for the expression limited to root-tip cells when tagged with 35Smini. This finding can be emphasized by the observation that the specific GAL4/GFP expression in J3411 was not replicated by the expression of pAt2G36360-3nGFP [33], because the authors, based on knowing of the same coding direction of the T-DNA and flanking gene At2G36360 (Fig. 1a, [33]), just took 3.9 kb fragment (not including our identified E_rtip1_) upstream of the At2G36360 coding start as a putative promoter to test its activity [33]. This leads to a speculation that the GAL4/GFP specific-expression in the root apex of J3411 might be attributed to regulatory elements located in the genomic DNA immediately upstream of the T-DNA insertion [33]. Regarding the possible target gene of the E_rtip1_, since it was documented that enhancers can be interacted with multiple transcription factors (TFs) to activate the transcription of genes located up to many kb or even several Mb away [41, 42], and can function in an orientation-independent manner [42], we propose that the E_rtip1_ might target to At2G36380 or At2G36360 or the both, being responsible for the specific GAL4/GFP expression pattern in J3411. Thus, it would be interesting to test the effect of the E_rtip1_ on specificity of the promoter in the root tips of these two genes in our future work.

### The specific activity of E_rtip1_ in the root apex is responsive to varied growth conditions

**Fig. 4:**
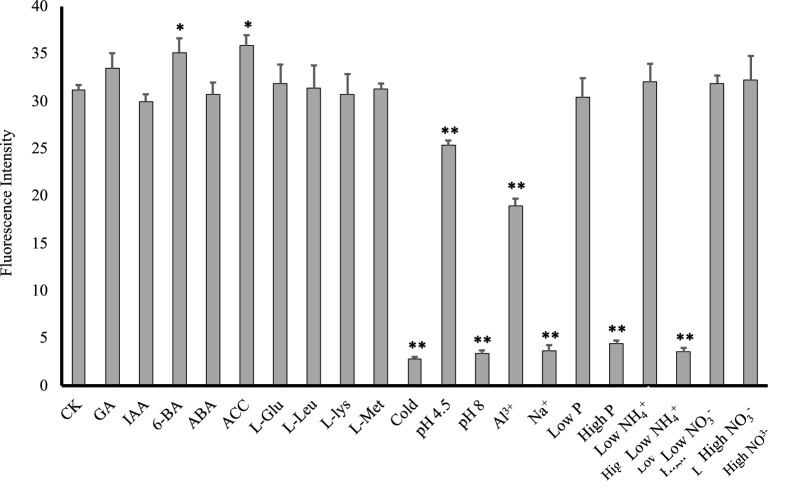
Effect of twenty different growth conditions on GFP-intensity in the root tip of P_Ertip1+35Smini_:*GFP*-expressing plants. Plants were pre-cultured vertically on medium agar-plates for 7 d and then transferred to the medium plates with 20 different treatments for 24 h growth (see “Methods” section). The treatments are shown below: IAA (60 nM), ABA (200 nM), GA (500 nM), ACC (500 nM), 6-BA (100 nM), l-Glu (0.5 mM), l-Leu (0.5 mM), l-Lys (0.5 mM), l-Met (0.5 mM), pH (4.5 and 8), P (phosphorus, high-2.5 mM, low-50 μM), NH_4_^+^ (high-10 mM, low-10 µM; in the form of (NH_4_)_2_SO_4_), NO_3_^−^ (high-10 mM, low-10 µM; in the form of KNO_3_), AlCl_3_ (50 μM), NaCl (80 mM), cold (4 °C). Control plants (CK) were grown on a half-strength of MS medium, which was also used as basic nutrients in the different treatment experiments (except for N- or P-treatment). All plants were cultivated under normal growth conditions, except for those treated with 4 °C. The intensity of green fluorescence photographed by using a fluorescence microscope-camera device was quantified using ImageJ (see “Methods” section). GA, gibberelin. 6-BA, 6-Benzylaminopurine (a type of cytokinins). ACC, 1-Aminocyclopropanecarboxylic acid (a precursor for ethylene biosynthesis). Mean values SE (n = 8) were potted. “*” or “**” indicates statistically significance calculated by using one-sided paired t test at P < 0.05 or P < 0.01

The root tip/apex represents a crucial part equipped with exquisite molecular processes and responsive to various signals from internal and soil environments, contributing to the ability of the plant to grow adaptively. To test the stability of the E_rtip1_ activity in the root tip, green fluorescence signals were measured in the P_Ertip1+35Smini_:*GFP*-containing plants, which were transferred for 24 h growth under 20 different conditions after 7 d pre-culture on normal agar-medium (Fig. 4, see “Methods” section). In the most cases tested (see “Methods” section. e.g. factors affecting the root growth, such as certain phytohormones, macro-nutrients and amino acids found to influence Arabidopsis root system formation in our recent study [9], and some abiotic stresses), the fluorescence intensity remained strong and stable in the root apex (Fig. 4), very similar to the control (CK, no treatment, Fig. 4). This property in terms of P_Ertip1+35Smini_ activity might be involved in the role of E_rtip1_-containing *cis*-acting regulatory elements i.e. OSE2ROOTNODULE, ROOTMOTIFTAPOX1 and DOFCOTEZM (Fig. 2b), which were reported to be critical for the abundant root-expression and tissue-/organ-specific transcription enhancement [37, 40, 43]. Under supply with 6-BA (a type of cytokinin) and ACC (a precursor for ethylene biosynthesis), the GFP expression was significantly up-regulated (Fig. 4), assuming that cytokinin and ethylene action-related *cis*-elements (AGCBOXNPGLB and GCCCORE, CPBCSPOR, Fig. 2b) in the E_rtip1_ might be molecular players but needed to be experimentally examined later.

**Fig. 5:**
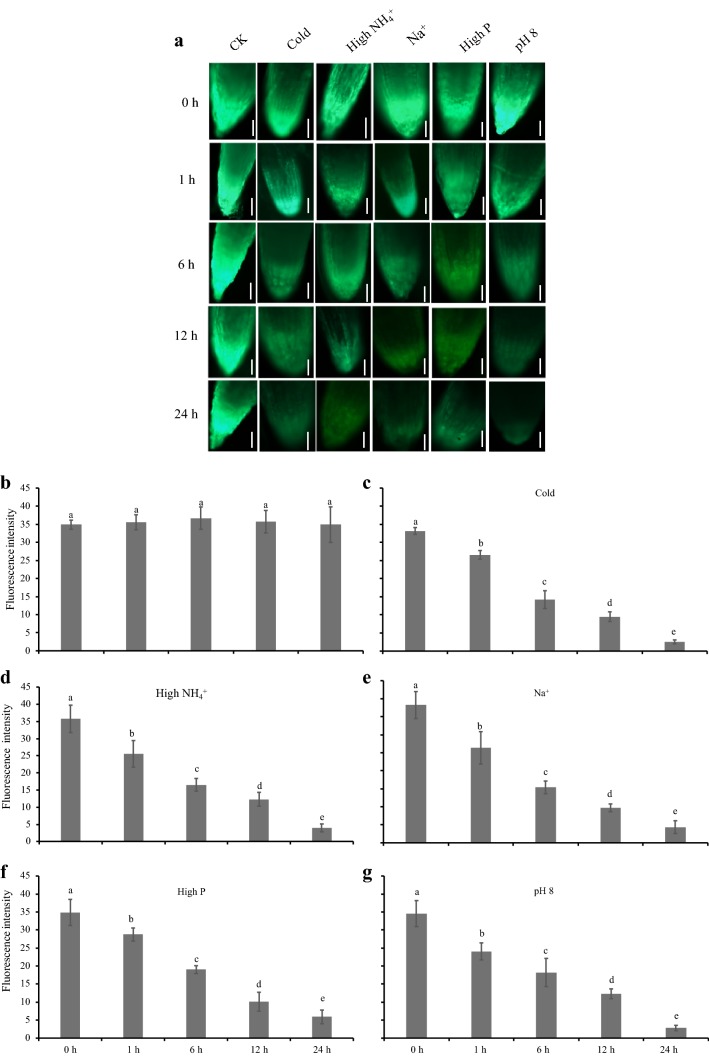
Down-regulation of E_rtip1_-dependent root-tip GFP expression by five environmental cues within 24 h. Growth of transgenic plants harboring P_Ertip1+35Smini_:*GFP*, microscopic observation and quantification of fluorescence signals derived from GFP are described as that in Fig. 4. **a** Representative image of green fluorescence signals from GFP in the root apex. Pictures were taken after exposure of plants to the treatments i.e. cold (4 °C), NH_4_^+^ (10 mM), salt (80 mM), P (2.5 mM) and pH 8 for 0 h, 1 h, 6 h, 12 h or 24 h. Bars = 50 μm. **b**–**g** Quantification of green fluorescence intensity in the root apex of plants without the treatment (CK) or with the treatment of cold (4 °C), NH_4_^+^ (10 mM), Na^+^ (80 mM), P (2.5 mM) or pH 8. Means of 6 biological replicates ± SD (n = 6) were plotted and different letters above the bars indicate statistically significant differences (*P* < 0.05 by one-way ANOVA and a multiple comparison test)

Remarkably, when subjected to certain stresses e.g. cold (4 °C), higher pH (8), salt (Na^+^, 80 mM), higher phosphorus (P, 2.5 mM) and ammonium (NH_4_^+^, 10 mM) in particular, the fluorescence intensity was strongly decreased by 10.9-, 8.9-, 8.4-, 7.2- and 8.7-fold, respectively (Fig. 4); besides, aluminium (Al^3+^, 50 µM) and low pH (4.5) also obviously (but not much strongly) supressed *GFP* transcription driven by the P_Ertip1+35Smini_ in the root apical area (Fig. 4). To further comprehend the sensitivity of P_Ertip1+35Smini_ activity in response to the above stress stimuli, fluorescence signals were quantified in a time depend manner (Fig. 5). Compared to the untreated plants, within 24 h the fluorescence intensity in the root tip was rapidly decreased by cold, salt, higher pH or P or NH_4_^+^, for instance, 1 h or 6 h treatment with these abiotic stresses could reduce GFP signals by approximately 25% and 50%, respectively (Fig. 5a, b). This intensive and fast repression of the fluorescence signals would indicate that the enhancer E_rtip1_ should contain some particular domains, which are required for down-regulation of the E_rtip1_ activity in the root apex of plants when subjected to such environmental stimuli tested. Since the plant (root) growth in response to varied external stresses and nutrients (including N and P) are largely modulated by the action of different phytohormones at a molecular level including a dramatic alteration of the gene expression [44, 45], several hormonal action-related domains (e.g. AGCBOXNPGLB, GCCCORE, CPBCSPOR, ABRE, TCA and GAREAE) predicted in the E_ript1_ might be putative *cis*-regulatory elements, which involve the activity reduction of this enhancer by the stress factors such as cold, salt, and higher P and N examined. In this study, although we have addressed on the characterisation of the overall activity and stability of the root tip specific enhancer E_rtip1_, the possible stress-responsive or regulatory elements embedding in this enhancer remain puzzled and are worthy to be studied in the future.

## Conclusion

The present work describes an uncomplicated and valuable process for the successful isolation and characterisation of a transcriptional enhancer E_rtip1_ specific for the root tip from an enhancer trap plant J3411. The root apex-specific activity of the E_rtip1_ was proven by the expression of both GFP and GUS reporters under the control of E_rtip1_ + 35Smini (functioning as a promoter P_Ertip1+35Smini_) in Arabidopsis. Interestingly, among 20 different growth conditions tested, the E_rtip1_ activity in the root tip cells remained stable in most cases, but was rapidly reduced under cold, salt, alkaline pH, higher ammonium and phosphorus. From an application viewpoint, we deem that the identified enhancer E_rtip1_ and its related synthetic promoter (e.g. P_Ertip1+35Smini_, Additional file 5: Fig. S2) should provide potent molecular means to favour comprehensive study and manipulation of any interested functional gene at the root apex, which may involve root-system formation and biological function in response to external and environmental cues.

## Additional files

**Additional file 1: Table S1.** Primers used in this study.

**Additional file 2: Table S2.** Putative *cis*-acting elements predicted in the enhancer E_rtip1_.

**Additional file 3: Table S3.** Summary of independent transgenic lines generated for each of five constructs.

**Additional file 4: Fig. S1.** Generation of independent transgenic lines of E_rtip1_ + 35Smini:GUS or :GFP. Three independent transgenic lines of E_rtip1_ + 35Smini:GUS show GUS expression in the root apex (a-c. Line a was used in the Fig. 3a–d). Six independent lines of E_rtip1_ + 35Smini:GFP exhibit GFP expression in the root tip region (d-h. Line d was used in the Fig. 3e). A bar scale = 100 μm.

**Additional file 5: Fig. S2.** The nucleotide sequence of E_rtip1_ + 35Smini. The uppercase letters and the letters underlined indicate respectively the 35S minimal promoter and 93 bp sequence from the T-DNA insert of J3411 line. The TATA-box in the 35Smini is boxed.

## Abbreviations

GFP: green fluorescent protein
GUS: β-glucuronidase
GAL4: a transcription factor positively regulating expression of galactose-induced genes
RB and LB: T-DNA right- and left-border
NPTII: a marker for bacterial kanamycin-resistance selection
UAS: GAL4 upstream activation sequence
IAA: indo-3-acetic acid
ABA: abscisic acid
GA: gibberellin
ACC: 1-aminocyclopropane-1-carboxylic acid
6-BA: 6-benzylaminopurine
CTAB: cetyltrimethyl ammonium bromide
FT: transcription factor
