# Supplementary material for: Molecular identification of a root apical cell-specific and stress-responsive enhancer from an Arabidopsis enhancer trap line
Source: Plant Methods. 2019 Jan 31;15:8. doi: 10.1186/s13007-019-0393-0 (PMC6354418; doi:10.1186/s13007-019-0393-0)
Supplement: Supplementary file 1 — Additional file 1: Table S1. Primers used in this study. [file 13007_2019_393_MOESM1_ESM.docx]

Table S1**:** Primers used in this study

| Primer name | Sequence (5'-3') |
| --- | --- |
| LongAd | GTAATACGACTCACTATAGGGCACGCGTGGTCGACGGCCCGGGCTGC |
| ShortHind | P-AGCTGCAGCCCG-NH2 |
| ShortEco | P-AATTGCAGCCCG-NH2 |
| AP1 | GTAATACGACTCACTATAGGGC |
| Lba1 | TGGTTCACGTAGTGGGCCATCG |
| AP2-C | TGGTCGACGGCCCGGGCTGC |
| LBb1 | GCGTGGACCGCTTGCTGCAACT |
| E_rtip1_-f | GAactagtTAGGAAACTATGCGATCAGA |
| TATA-r | AGactagtTGTTGGATCCGGTTCTCT |
| E_rtip1_-r | AGactagtAGAAAGCTGTACTCATTATA |
| E_rtip-2_-f | ATactagtGATTTTTATATACATGGACG |
| E_rtip-2_-r | AGactagTTTCATTTTTTTAGGGAAAGG |

Note: LongAd, ShortHind, ShortEco, AP1, Lba1, AP2-C, LBb1 for Nest PCR (according to the protocol from http://signal.salk.edu/T-DNArecovery.pdf). Other primers used for the amplification and further cloning of five putative enhancer/promoter sequences from J3411 line: E_rtip1_-f and E_rtip1_-r for *E_rtip1_*, E_rtip1_-f and TATA-r for *E_rtip1_+35Smini*, E_rtip-2_-f and E_rtip1_-r for *E_rtip2_*, E_rtip2_-f and TATA-r for *E_rtip2_+35Smini,* E_rtip-1_-f and E_rtip2_-r for *E_rtip3_.* Lowercase letters in the primes indicate a restriction site for cloning.
