# Supplementary material for: Molecular identification of a root apical cell-specific and stress-responsive enhancer from an Arabidopsis enhancer trap line
Source: Plant Methods. 2019 Jan 31;15:8. doi: 10.1186/s13007-019-0393-0 (PMC6354418; doi:10.1186/s13007-019-0393-0)
Supplement: Supplementary file 2 — Additional file 2: Table S2. Putative cis-acting elements predicted in the enhancer Ertip1. [file 13007_2019_393_MOESM2_ESM.docx]

Table S2. Putative *cis*-acting elements predicted in the enhancer E_rtip1_.

| cis-element | Consensus | Number of repeats | Position | Role | Reference |
| --- | --- | --- | --- | --- | --- |
| ABRELATERD1 | ACGTG | 2 | -1543, -84 | etiolation-induced, responsive to dehydration | [1] |
| ABRE | CACG | 1 | -1760 | abscisic acid responsiveness | [2] |
| ACGTATERD1 | ACGT | 9 | -1811, -1543, -1039, -857, -850, -787, -536, -185 | etiolation-induced, responsive to dehydration | [1] |
| AGCBOXNPGLB | AGCCGCC | 1 | -599 | Ethylene-responsive, negative response to salt stress | [3, 4] |
| ARR1AT | NGATT | 7 | -1900, -1757, -1564, -1459, -1317, -947, -381 | Response regulator | [5] |
| CAATBOX1 | CAAT | 6 | -1414, -1180, -991, -588, -319, +4 | Tissue specific expression | [6] |
| CPBCSPOR | TATTAG | 2 | -957, -752 | Cytokinin-responsive | [7] |
| DOFCOREZM | AAAG | 8 | -1933, -1915, -1622, -635, -524, -408, -195, -97 | Enhances transcription | [8] |
| GCCCORE | GCCGCC | 1 | -598 | ethylene-responsive | [9] |
| GAREAE | TAACAGA | 1 | -679 | gibberellin-responsiveness | [10] |
| MYCCONSENSUSAT | CANNTG | 8 | -1960, -1717, -1528, -1246, -1048, -808, -426, -335 | Drougt, cold，ABA-responsive | [11, 12] |
| OSE2ROOTNODULE | CTCTT | 2 | -1807, -1801 | Root-specific expression | [13] |
| ROOTMOTIFTAPOX1 | ATATT | 5 | -1875, -1076, -998, -879, -472 | Root-specific expression | [14] |
| TATA-BOX | TACA  TAAA  TAGA  TATA | 21 | -1695, -1665, -1592, -1572, -1533, -1433, -1431, -1429, -1423, -1407, -1253, -747, -482, -440, -438, -419, -417, -313, -155, -151, +50 | core promoter element around -30 of transcription start | [15] |
| TATAPVTRNALEU | TTTATATA | 2 | -942, -420 | Transcription re-initiation | [3] |
| TATC | TATCCA | 1 | -1612 | gibberellin-responsiveness | [16] |
| TCA-element | CCATCTTTTT | 1 | -1923 | salicylic acid responsiveness | [17] |

1. Kuroi T, Nakamoto K. Two different novel cis-acting elements of erd1, a clpA homologous Arabidopsis gene function in induction by dehydration stress and dark-induced senescence. Plant J. 2003;33:259-70.

2. Guiltinan MJ, Marcotte WR, Quatrano RS. A plant leucine zipper protein that recognizes an abscisic acid response element. Science. 1990;250:267-71.

3. Yukawa Y, Sugita M, Choisne N, Small I, Sugiura M. The TATA motif, the CAA motif and the poly(T) transcription termination motif are all important for transcription re-initiation on plant tRNA genes. Plant J. 2000;22:439–47.

4. Wang L, Wang C, Qin L, Liu W, Wang Y. ThERF1 regulates its target genes via binding to a novel cis-acting element in response to salt stress. Journal of Integrative Plant Bio. 2015;57:838-47.

5. Sakai H, Aoyama T, Oka A. Arabidopsis ARR1 and ARR2 response regulators operate as transcriptional activators. Plant J. 2000;24:703-11.

6. Shirsat A, Wilford N, Croy R, Boulter D. Sequences responsible for the tissue specific promoter activity of a pea legumin gene in tobacco. Mol Gen Genet. 1989;215:326.

7. Fusada N, Masuda T, Kuroda H, Shimada H, Ohta H, Takamiya K. Identification of a novel cis-element exhibiting cytokinin-dependent protein binding in vitro in the 5'-region of NADPH-protochlorophyllide oxidoreductase gene in cucumber. Plant Mol Biol. 2005;59:631-45.

8. Yanagisawa S, Schmidt RJ. Diversity and similarity among recognition sequences of Dof transcription factors. Plant J. 1999;17:209.

9. Chakravarthy S, Tuori RP, D'Ascenzo MD, Fobert PR, Despres C, Martin GB. The tomato transcription factor Pti4 regulates defense-related gene expression via GCC box and non-GCC box cis elements. Plant Cell. 2003;15:3033-50.

10. Sutoh K, Yamauchi D. Two cis-acting elements necessary and sufficient for gibberellin-upregulated proteinase expression in rice seeds. Plant J. 2003;34:635-45.

11. Abe H, Urao T, Ito T, Seki M, Shinozaki K, Yamaguchi-Shinozaki K. Arabidopsis AtMYC2 (bHLH) and AtMYB2 (MYB) Function as Transcriptional Activators in Abscisic Acid Signaling. Plant Cell. 2003;15:63.

12. Bos A, Ingvarsson P. Nucleotide polymorphism in ICE1 : a regulator of cold induced freezing tolerance in Arabidopsis thaliana. 2008.

13. Stougaard J, Sandal NN, Grøn A, Kühle A, Marcker KA. 5' Analysis of the soybean leghaemoglobin lbc(3) gene: regulatory elements required for promoter activity and organ specificity. EMBO J. 1987;6:3565-9.

14. Elmayan T, Tepfer M. Evaluation in tobacco of the organ specificity and strength of the rolD promoter, domain A of the 35S promoter and the 35S 2 promoter. Transgenic Res. 1995;4:388-96.

15. Grace ML, Chandrasekharan MB, Hall TC, Crowe AJ. Sequence and spacing of TATA box elements are critical for accurate initiation from the beta-phaseolin promoter. J Biol Chem. 2004;279:8102.

16. Isabel-Lamoneda I, Diaz I, Martinez M, Mena M, Carbonero P. SAD: a new DOF protein from barley that activates transcription of a cathepsin B‐like thiol protease gene in the aleurone of germinating seeds. Plant J. 2003;33:329-40.

17. Hennig J, Dewey RE, Cutt JR, Klessig DF. Pathogen, salicylic acid and developmental dependent expression of a beta-1,3-glucanase/GUS gene fusion in transgenic tobacco plants. Plant J. 1993;4:481-93.
