# Supplementary material for: Molecular identification of a root apical cell-specific and stress-responsive enhancer from an Arabidopsis enhancer trap line
Source: Plant Methods. 2019 Jan 31;15:8. doi: 10.1186/s13007-019-0393-0 (PMC6354418; doi:10.1186/s13007-019-0393-0)
Supplement: Supplementary file 3 — Additional file 3: Table S3. Summary of independent transgenic lines generated for each of five constructs. [file 13007_2019_393_MOESM3_ESM.docx]

| Construct | GUS-expressed lines/total lines | GFP-expressed lines/total lines |
| --- | --- | --- |
| E_rtip1_:reporter | 0/4 | 0/6 |
| E_rtip1_+35Smini:reporter | 3/3 | 6/6 |
| E_rtip2_:reporter | 0/5 | 0/4 |
| E_rtip2_+35Smini:reporter | 0/4 | 0/9 |
| E_rtip3_:reporter | 0/2 | 0/5 |

Table S3. Summary of independent transgenic lines generated for each of five constructs.

Note: “reporter” indicates either *GUS* or *GFP*. All created independent lines (at least two for each construct) were selected for their kanamycin resistance till T_2_-T_3_ generation. The expression or activity of the reporter was examined in different plant tissues/organs, of which only the root tip showed the occurrence of GUS or GFP in the “E_rtip1_+35Smini:reporter” lines. The reporter expression was not observed in any tissue/organ of the lines harboring the rest four constructs.
