# Supplementary material for: Molecular identification of a root apical cell-specific and stress-responsive enhancer from an Arabidopsis enhancer trap line
Source: Plant Methods. 2019 Jan 31;15:8. doi: 10.1186/s13007-019-0393-0 (PMC6354418; doi:10.1186/s13007-019-0393-0)
Supplement: Supplementary file 4 — Additional file 4: Fig. S1. Generation of independent transgenic lines of Ertip1 + 35Smini:GUS or :GFP. Three independent transgenic lines of Ertip1 + 35Smini:GUS show GUS expression in the root apex (a-c. Line a was used in the Fig. 3a–d). Six independent lines of Ertip1 + 35Smini:GFP exhibit GFP expression in the root tip region (d-h. Line d was used in the Fig. 3e). A bar scale = 100 μm. [file 13007_2019_393_MOESM4_ESM.pdf]

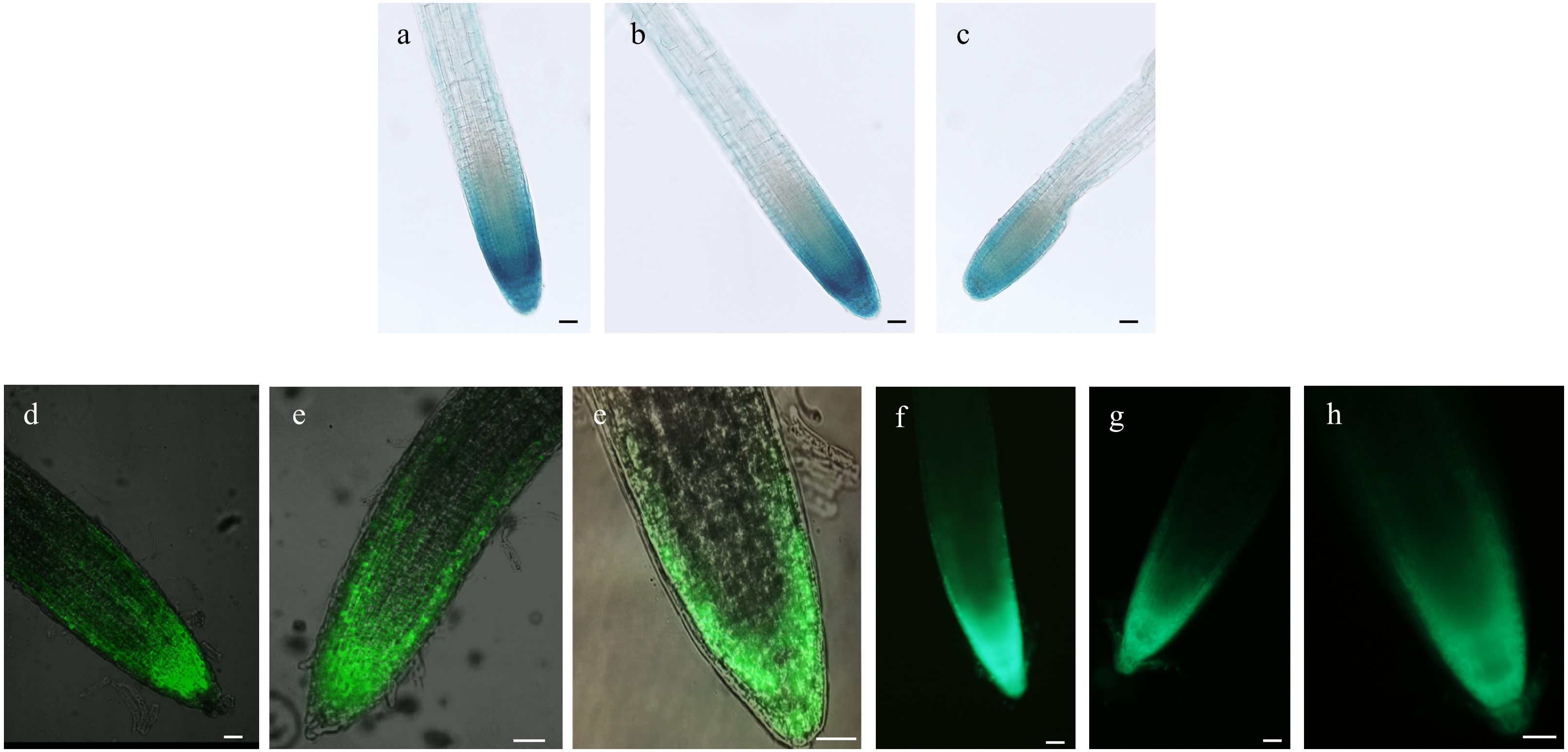

Fig S1. Generation of independent transgenic lines of E_{rtip1}+35Smini:GUS or :GFP. Three independent transgenic lines of E_{rtip1}+35Smini:GUS show GUS expression in the root apex (a-c. Line a was used in the Fig 3a-d). Six independent lines of E_{rtip1}+35Smini:GFP exhibit GFP expression in the root tip region (d-h. Line d was used in the Fig 3e). A bar scale=100 μm.
