# Supplementary material for: Molecular identification of a root apical cell-specific and stress-responsive enhancer from an Arabidopsis enhancer trap line
Source: Plant Methods. 2019 Jan 31;15:8. doi: 10.1186/s13007-019-0393-0 (PMC6354418; doi:10.1186/s13007-019-0393-0)
Supplement: Supplementary file 5 — Additional file 5: Fig. S2. The nucleotide sequence of Ertip1 + 35Smini. The uppercase letters and the letters underlined indicate respectively the 35S minimal promoter and 93 bp sequence from the T-DNA insert of J3411 line. The TATA-box in the 35Smini is boxed. [file 13007_2019_393_MOESM5_ESM.pdf]

```
   1 gacaccccta ggaaactatg cgatcagaaa taatatatac agatgctata atctgctttc
  61 aaatacaaag cactaaactg agacaaagca tatctttgaa gattccaaac tttgaagggt
 121 atccatatta ttattcgata atgttgaact tacttttgtt ttttttttat ctttgcgtac
 181 aatttggaac gtctcttact cttttgctat tgaagtttga atttcatata acatacaaaa
 241 tgagatttat gttaacatgg gcacactcta acagtagaaa tacatatgca taatataatt
 301 cacttataag tatatggtgc tttaatagaa gctggtagtt gaaaatttga aggctatttt
 361 taacagaaat aaattctaaa gactattttg agtaaaaatc attcataggg cgatctatat
 421 atagtaccat atagatgatt gtgagataat caatgaacgt gagtagagct acacatgttt
 481 gaatgagaat atcggacggt cagaaataaa cggaagagat atcgcaaccc acgaagtaag
 541 ggattttcat cacatgacaa cagtcatcaa tttagctatt ccctatgcat tccattaata
 601 cgataatgaa tggtgtgtgg catcatcata accacaatca ttaattaata ataccttaat
 661 aatcaaatac gatctttttt taagatttag agagttccca aaatgtttaa ttattttaaa
 721 atttaatatag gtaaaaattt gcaggagaac atccatgtgc tcaacaaatt ggtgagaggt
 781 tttgttgttt cttttatttt tgtttaagtt ctctctattt atcgtatttg tgctaagcgc
 841 ttaactcatc gaataagctt tggataattc agaacctaaa cttttgacat taccagttat
 901 aatttaatcc aaataatgac aaaatattat atctagttta atttaaaaca acacatgcac
 961 acgtaatgta tggcgtcacc tatgacgaat tgtgttttct tatatttatt ctttcctttc
1021 cctaaaaaaa tgaatagagc gttattagtt tatgattttt atatacatgg acggttgcat
1081 gacatttttt ttattacccg ttaaaacagt gtcattcatc atatttgacc tgtcaaaaac
1141 tcacgtagca cgttacaaat atatatatgt atagggtccc aatcatataa acagatgcga
1201 tatgcatggc tcacgttctt ggaacgctag agtttttttt gaagttttat tagttattga
1261 taatttctcc aactctaact tttttatct tcttttacat aaattatctg ctgctgtaaa
1321 taacaaattt tttgggtcgt atactcgaat ttccatcaaa accaaaagtt ctgaaaaaat
1381 cccacaatat ataatatata agccgccaag agccaattcc atcttgtgga tcactaaacc
1441 agtatttttg gggtttgaat ctcacgttct ccattaaaga ataaggacat gcaagtaagc
1501 aaaaacaagt tccatataag cagaaccata ttccggagga ctgcaacttt caaaatgtaa
1561 tatatagcgg aatcatctgt ttatatacag aaaagtaggg ttcaagcctt tcgacttcag
1621 attttttttt aaaatttac tttagaactt caatcataat taagcacatg taaacggctt
1681 accaatttat aattaaaaaa aaaaaacggt cattgtgctg aaaaatatca gcgtttttat
1741 tttttagtat gtgtacaaga caattttaag tggatcgttt actaaacgag agatgtccct
1801 gtgaaaagct gtccacgtac gaaattgatc tgtccccaac ggccacctat ataaaattga
1861 actaaacctt aattacttta ttggtttgtt gctttaatta ctaaagttag tagggacgtg
1921 actattacgg gttttctttt cttttcccaa aaccttaatt gctaaaatca cattctaatg
1981 tataatgagt acagctttct cccgccaata tatcctgtca aacactggat CTTCGCAAGA
2041 CCCTTCCTCT ATATAAGGAA GTTCATTTCA TTTGGAGAGA Accggatcca aca
                  TATA-box
```

Fig S2. The nucleotide sequence of E_{rtip1}+35Smini. The uppercase letters and the letters underlined indicate respectively the 35S minimal promoter and 93 bp sequence from the T-DNA insert of J3411 line. The TATA-box in the 35Smini is boxed.
